# Observability and Controllability of Nonlinear Networks: The Role of Symmetry

**DOI:** 10.1103/PhysRevX.5.011005

**Published:** 2015-01-23

**Authors:** Andrew J. Whalen, Sean N. Brennan, Timothy D. Sauer, Steven J. Schiff

**Affiliations:** Department of Mechanical and Nuclear Engineering, and Center for Neural Engineering, The Pennsylvania State University, University Park, Pennsylvania 16802, USA; Department of Mechanical and Nuclear Engineering, and Center for Neural Engineering, The Pennsylvania State University, University Park, Pennsylvania 16802, USA; Department of Mathematical Sciences, George Mason University, Fairfax, Virginia 22030, USA; Departments of Engineering Science and Mechanics, Neurosurgery and Physics, Center for Neural Engineering, The Pennsylvania State University, University Park, Pennsylvania 16802, USA

**Keywords:** Biological Physics, Complex Systems, Nonlinear Dynamics

## Abstract

Observability and controllability are essential concepts to the design of predictive observer models and feedback controllers of networked systems. For example, noncontrollable mathematical models of real systems have subspaces that influence model behavior, but cannot be controlled by an input. Such subspaces can be difficult to determine in complex nonlinear networks. Since almost all of the present theory was developed for linear networks without symmetries, here we present a numerical and group representational framework, to quantify the observability and controllability of nonlinear networks with explicit symmetries that shows the connection between symmetries and nonlinear measures of observability and controllability. We numerically observe and theoretically predict that not all symmetries have the same effect on network observation and control. Our analysis shows that the presence of symmetry in a network may decrease observability and controllability, although networks containing only rotational symmetries remain controllable and observable. These results alter our view of the nature of observability and controllability in complex networks, change our understanding of structural controllability, and affect the design of mathematical models to observe and control such networks.

## I. INTRODUCTION

An observer model of a natural system has many useful applications in science and engineering, including understanding and predicting weather or controlling dynamics from robotics to neuronal systems [1]. A fundamental question that arises when utilizing filters to estimate the future states of a system is how to choose a model and measurement function that faithfully captures the system dynamics and can predict future states [2,3]. An observer is a model of a system or process that assimilates data from the natural system being modeled [4] and reconstructs unmeasured or inaccessible variables. In linear systems, the key concept to employ a well-designed observer is observability, which quantifies whether there is sufficient information contained in the measurement to adequately reconstruct the full system dynamics [5,6].

An important problem when studying networks is how best to observe and control the entire network when only limited observation and control input nodes are available. In classic work, Lin [7] described the topologies of graph directed linear networks that were structurally controllable. Incorporating Lin’s framework, Liu et al. [8] described an efficient strategy to count the number of control points required for a complex network, which have an interesting dependence on time constant [9]. Structural observability is dual to structural controllability [10]. In Ref. [11], the requirements of structural observability incorporated explicit use of transitive components of directed graphs—fully connected subgraphs where paths lead from any node to any other node—to identify the minimal number of sites required to observe from a network.

All of these prior works depend critically on the dynamics being linear and generic, in the sense that network connections are essentially random. Joly [12] showed that transitive generic networks with nonlinear nodal dynamics are observable from any node. Nevertheless, symmetries are present in natural networks, as evident from their known structures [13] as well as the presence of synchrony. Recently, Golubitsky et al. [14] proved the rigid phase conjecture—that the presence of synchrony in networks implies the presence of symmetries and vice versa. In particular, synchrony is an intrinsic component of brain dynamics in normal and pathological brain dynamics [15].

Our present work is motivated by the following question: What role do the symmetries and network coupling strengths play when reconstructing or controlling network dynamics? The intuition here is straightforward: consider three linear systems with identical dynamics [diagonal terms of the system matrix *A* in $\dot{x}(t)=Ax\text{(}t\text{)]}\text{.}$ If the coupling terms are identical (off-diagonal terms of *A*), it is easy to show that the resulting observability of individual states becomes degenerate as the rows and columns of the system matrix become linearly dependent under elementary matrix operations. For example, consider the trivial case of a 3 × 3 system matrix of ones:

$$\dot{x}=Ax\text{=}\left[\begin{array}{ccc} 1 & 1 & 1\\ 1 & 1 & 1\\ 1 & 1 & 1 \end{array}\right]\;\;[\begin{array}{c} x_{1}\\ x_{2}\\ x_{3} \end{array}]\;.\;\;$$

The system is degenerate in the sense that there is only one dynamic, as the rows and columns of *A* are not independent. This lack of independent rows and columns of the system matrix has direct implications for the controllability and observability of the system. For example, in this trivial system, the difference between any two of the states is constrained to a constant *x*_1_ – *x*_2_ = *c*; thus, there is no input coupled to the third state *x*_3_ that could control both *x*_1_ and *x*_2_ independently from each other. Taking a single measurement in Eq. (1), *y*_= [1, 0, 0]**x**, the system is not_ observable; however, taking an additional measurement, the system is fully observable. The details of this computation will be explained in detail in the following section.

$$y=\left[\begin{array}{l} 1,0,0\\ 0,1,0 \end{array}\right]x\text{,}$$

In fact, for the more general case of linear time-varying networks, group representation theory [16] has been utilized to show that linear time-varying networks can be noncontrollable or nonobservable due to the presence of symmetry in the network [17]. Brought into context, in networks with symmetry, Rubin and Meadows [17] defined a coordinate transform that decomposes the network into decoupled observable (controllable) and unobservable (uncontrollable) subspaces, which can then be determined by inspection like our previous trivial example. Recently, Pecora *et al*. [18] utilized this same method to show how separate subsets of complex networks could synchronize and desynchronize according to these same symmetry-defined subspaces. Interestingly, while Ref. [17] has been a rather obscure work, it is based on Wigner’s work in the 1930s applying group representation theory to the mechanics of atomic spectra [19]. Thus, just as the structural symmetry of the Hamiltonian can be used to simplify the solution to the Schrodinger equation [20], the topology of the coupling in a network can have a profound impact on its observation and control.

In this article, we extend the exploration of observability and controllability to network motifs with explicit nonlinearities and symmetries. We further explore the effect of coupling strength within such networks, as well as spatial and temporal effects on observability and controllability. Lastly, we demonstrate the utility of the linear analysis of group representation theory as a tool with which to gain insights into the effects of symmetry in nonlinear networks. Our findings apply to any complex network, including power grids, the internet, genomic and metabolic networks, food webs, electronic circuits, social organization, and brains [8,11,18,21].

## II. BACKGROUND

From the theories of differential embeddings [22] and nonlinear reconstruction [23,24] we can create a nonlinear measure of observability composed of a measurement function and its higher Lie derivatives employing the differential embedding map [25]. The differential embedding map of an observer provides the information contained in a given measurement function and model, which can be quantified by an index [26–28]. Computed from the Jacobian of the differential embedding map, the observability index is a matrix condition number that quantifies the perturbation sensitivity (closeness to singularity) of the mapping created by the measurement function used to observe the system. There is a dual theory for controllability, where the differential embedding map is constructed from the control input function and its higher Lie brackets with respect to the nonlinear model function [29,30]. Singularities in the map cause information about the system to be lost and observability to decrease. Additionally, the presence of symmetries in the system’s differential equations makes observation difficult from variables around which the invariance of the symmetry is manifested [31,32]. We extend this analysis to networks of ordinary differential equations and investigate the effects of symmetries on observability and controllability of such networks as a function of connection topology, measurement function, and connection strength.

### A. Linear observability and controllability

In the early 1960s, Kalman introduced the notions of state space decomposition, controllability, and observability into the theory of linear systems [5]. From this work comes the classic concept of observability for a linear time-invariant dynamic system, which defines a “yes” or “no” answer to the question of whether a state can be reconstructed from a measurement using a rank condition check.

A dynamic model for a linear (time-invariant) system can be represented by

$$\dot{x}\;\;(t)=Ax\;(t)+Bu(t),\;$$

y(t)=Cx​ (t),

where $x\in {\mathbb{R}}^{n}$ represents the state variable, $u\in {\mathbb{R}}^{m}$ is the external input to the system, and $y\in {\mathbb{R}}^{p}$ is the output (measurement) function of the state variable. Typically, there are less measurements than states, so *p* < *n*. The intuition for observability comes from asking whether an initial condition can be determined from a finite period of measuring the system dynamics from one or more sensors. That is, given the system in Eq. (2), with **x** (*t*) = *e*^*At*^ **x**_**0**_ and *B***u**=0, determine the initial condition **x**_**0**_ from measurement y(t),0≤t≤T. To evaluate this locally, we take higher derivatives of *y*(*t*):

(3) y(t)=Cx(t)  

$$\dot{y}=C\dot{x}(t)=CAx\text{(}t\text{)}\;$$

$$\ddot{y}=CA\dot{x}(t)=CA^{2}x\text{(}t\text{)}\;\;\;\;$$

$$\begin{array}{l} \vdots \end{array}$$

$$y^{(}{{}^{n-1}}^{)}=CA^{n-1}x\text{(}t\text{)}.\;\;\;$$

Factoring the **x** terms and putting *y* and its higher derivatives in matrix form, we have a mapping from outputs to states

$$\left[\begin{array}{c} y\\ \dot{y}\\ \ddot{y}\\ \vdots \\ y^{\left(n-1\right)} \end{array}\right]=\left[\begin{array}{c} C\\ CA\\ CA^{2}\\ \vdots \\ CA^{n-1} \end{array}\right]x\text{,}$$

where the linear observability matrix [33] is defined as

(5) $$O\equiv \left[\begin{array}{c} C\\ CA\\ CA^{2}\\ \vdots \\ CA^{n-1} \end{array}\right].$$

The finite limit of taking derivatives in Eq.(3) comes from the Cayley-Hamilton theorem, which specifies that any square matrix A satisfies its own characteristic equation, which is the polynomial,p(λ)=0, where$p(\lambda )=\det(\lambda I_{n}-A)$.In other words, An is spanned by the lower powers of A, from $A^{0}$ to $A^{n-1}$,

$$y(t)=Ce^{At}x_{0,}\;\;\;\;\;\;with\;\;\;\;\;\;e^{At}\equiv \sum_{k=0}^{n-1}\alpha_{k}(t)A^{k},$$

$$\begin{array}{l} y(t)=[\alpha_{0}(t)C+\alpha_{1}(t)CA+\alpha_{2}(t)CA^{2} \end{array}$$

$$\;\;\;\;\;\;\;\;\;\;\;\;+\cdots +\alpha_{n-1}(t)CA^{n-1}]x_{0}$$

Thus, if the observability matrix spans *n* space [rank(*O*)=*n*], the initial condition *x*_0_ can be determined, as the mapping $x_{0}={\left(0^{T}0\right)}^{-1}0^{T}y(t)$ from output to states exists and is unique. More formally, the system Eq. (2) is locally observable (distinguishable at a point *x*_0_) if there exists a neighborhood of *x*_0_ such that $x_{0}\neq x_{1}\Rightarrow y(x_{0})\neq x_{1}\Rightarrow y(x_{1})$

In a similar fashion, the linear controllability matrix is derived from asking whether an input **u**(*t*)can be found to take any initial condition **x**(0)=*x*_0_ to arbitrary position **x**(*T*)=*x*_*f*_ in a finite period of time *T*. For the sake of simplicity, we assume a single input *u*(*t*) and take the higher derivatives of $\dot{x}\text{(}t\text{)=}Ax\text{(}t)+Bu(t)$ up to the (*n* – 1)the derivative of *u*(*t*) (again using the Cayley-Hamilton theorem):

$$\dot{x}\text{(}t\text{)=}Ax\text{(}t)+Bu(t)\;$$

$$\ddot{x}\text{(}t\text{)=}A^{2}x\text{(}t)+ABu(t)+B\dot{u}(t)\;$$

$$\dddot{x}\text{(}t\text{)=}A^{3}x\text{(}t)+A^{2}Bu(t)+AB\dot{u}(t)+B\ddot{u}(t)\;$$

$$\begin{array}{l} \vdots \end{array}$$

$$x^{\left(n\right)}\text{(}t\text{)=}A^{n}x\text{(}t)+A^{n-1}Bu(t)+A^{n-2}B\dot{u}(t)+\cdots \;$$

$$\;\;\;\;\;\;\;\;\;\;\;\;\;\;\;\;+Bu^{\left(n-1\right)}(t),$$

which gives us a mapping from input to states

$$\left[\begin{array}{c} \dot{x}(t)\\ \ddot{x}(t)\\ \vdots \\ x^{\left(n-1\right)}(t)\\ x^{\left(n\right)}(t) \end{array}\right]-\left[\begin{array}{c} A\\ A^{2}\\ \vdots \\ A^{\left(n-1\right)}\\ A^{\left(n\right)} \end{array}\right]x(t)=Q\left[\begin{array}{c} u(t)\\ \dot{u}\left(t\right)\\ \vdots \\ u^{\left(n-2\right)}(t)\\ u^{\left(n-1\right)}(t) \end{array}\right]$$

where the linear controllability matrix is defined [33] as

$$Q\equiv \left[B,AB,A^{2}B\cdots ,A^{n-1}B\right].$$

### B. Differential embeddings and nonlinear observability

From early work on the nonlinear extensions of observability in the 1970s [29,30], it was shown that the observability matrix for nonlinear systems could be expressed using the measurement function and its higher-order Lie derivatives with respect to the nonlinear system equations. The core idea is to evaluate a mapping ϕfrom the measurements to the states $\phi :{\mathbb{R}}^{P}\to {\mathbb{R}}^{n}$. In particular, Hermann and Krener [30] showed that the space of the measurement function is embedded in ${\mathbb{R}}^{n}$when the mapping from measurement to states is everywhere differentiable and injective by the Whitney embedding theorem [22,23]. An embedding is a map involving differential structure that does not collapse points or tangent directions [24]; thus, a map ϕ is an embedding when the determinant of the map Jacobian det $\left(\partial \phi /{\partial x|}_{\forall x\in {\mathbb{R}}^{n}}\right)$ is nonvanishing and one to one (injective). In a recent series of papers [25,28,31], Letellier *et al*. computed the nonlinear observability matrices for the well-known Lorenz and Rössler systems [34,35] and demonstrated that the order of the singularities present in the observability matrix (and thus the amount of intersection between the singularities and the phase space trajectories) was related to the decrease in observability. It is worth noting that the calculation of the observability matrix and locally evaluating the conditioning of the matrix over a state trajectory is a straightforward process and much more tractable than analytically determining the singularities (and thus their order) of the observability matrix of a system of arbitrary order. The former is limited only by computational capacity and the differentiability of the system equations to order *n* − 1, where *n* is the order of the system.

For a nonlinear system, we replace *A***x**(*t*) in Eq. (2) by a nonlinear vector field A_NL_(**x**(*t*)) and assume that the smooth scalar measurement function is taken as *y*(*t*) = *C***x** (*t*) and the system equations comprise the nonlinear vector field **f**(**x**(*t*)) = *A*_NL_(**x**(*t*)) (note that if there is no external input, then *B***u**(*t*) = 0, which we assume here to simplify the display of equations). (If B**u** ≠ 0, then as long as the input is known the mapping from output to states can be solved, and the determination of observability still relies on the conditioning of the matrix *O*.) As in the linear case, we evaluate locally by taking the higher Lie derivatives of *y*(*t*), and for compactness of notation, dependence on *t* is implied:

$$L_{f}^{0}(y(x))=y(x)\;$$

$$L_{f}^{1}(y(x))=\nabla y(x)f(x)=\frac{\partial y(x)}{\partial x}\cdot f(x)\;$$

$$L_{f}^{2}(y(x))=\frac{\partial }{\partial x}[L_{f}^{1}(y(x))]\cdot f(x)\;\;\;\;\;$$

$$\begin{array}{l} \;\vdots \end{array}$$

$$L_{f}^{k}(y(x))=\frac{\partial }{\partial x}[L_{f}^{k-1}(y(x))]\cdot f(x),$$

where **𝔏**_*f*_(*y*(*x*)) is the Lie derivative of ***y***(*x*) along the vector field **f**(*x*). More explicitly, we have $x\in {\mathbb{R}}^{n}$, so as a vector example, the first Lie derivative will take the form

$$L_{f}^{1}(y(x))=\left[\frac{\partial y(x)}{\partial x_{1}}\cdots \frac{\partial y(x)}{\partial x_{n}}\right]\cdot \left[\begin{array}{c} f_{1}(x)\\ \vdots \\ f_{n}(x) \end{array}\right]\cdot$$

With formal definitions of the measurement (output) function Eq. (2) and its higher Lie derivatives Eq. (5), the differential embedding map 𝜙 is defined as the Lie derivatives$L_{f}^{0}(y(x))\cdots L_{f}^{n-1}(y(x))$, where the superscripts represent the order of the Lie derivative from 0 to *n* − 1, where *n* is the order of the system A_NL_(**x**)

$$\phi =\left[\begin{array}{c} L_{f}^{0}(y(x))\\ L_{f}^{1}(y(x))\\ \vdots \\ L_{f}^{n-1}(y(x)) \end{array}\right]\cdot$$

Taking the Jacobian of the map ϕ, we arrive at the observability matrix

$$O\equiv \frac{\partial \phi }{\partial x}=\left[\begin{array}{ccc} \frac{\partial L_{f}^{0}(y(x))}{\partial x_{1}} & \cdots & \frac{\partial L_{f}^{0}(y(x))}{\partial x_{n}}\\ \vdots & \ddots & \vdots \\ \frac{\partial L_{f}^{n-1}(y(x))}{\partial x_{1}} & \cdots & \frac{\partial L_{f}^{n-1}(y(x))}{\partial x_{n}} \end{array}\right],$$

which reduces to Eq. (4) for linear system representations. The key intuition here is that in the nonlinear case the observability matrix becomes a function of the states, where a linear system is always a constant matrix of parameters.

### C. Lie brackets and Nonlinear controllability

The nonlinear controllability matrix is developed in Ref. [29] from intuitive control problem examples and given rigorous treatment in Ref. [30]; in a dual fashion to observability, the controllability matrix is a mapping constructed from the input function and its higher-order Lie brackets. The Lie bracket is an algebraic operation on two vector fields **f**(*x*), **g**(*x*) ∈ ℝ^n^ that creates a third vector field **𝔉**(*x*), which when taken with g as the input control vector **u** ∈ ℝ^m^ defines an embedding in ℝ^n^ that maps the input to states [30].

For a nonlinear system, we replace A**x**(*t*) in Eq. (2) by a nonlinear vector field *A*NL(**x**(*t*)), take the input function as *g*=*B***u**(*t*) in system Eq. (2), and create Lie brackets with respect to the nonlinear vector field **f**(**x**(*t*)) = *A*_NL_(**x**(*t*)). The Lie bracket is defined as

$$\left(ad_{f}^{1},g\right)=\left[f\text{,}g\right]=\frac{\partial g}{\partial x}f-\frac{\partial f}{\partial x}g\;$$

$$\left(ad_{f}^{2},g\right)=\left[f,\left[f,g\right]\right]=\frac{\partial \left(ad_{f}^{1},g\right)}{\partial x}f-\frac{\partial f}{\partial x}\;\left(ad_{f}^{1},g\right)\;$$

$$\begin{array}{l} \vdots \end{array}$$

$$\left(ad_{f}^{k},g\right)=\left[f\text{,}\left(ad_{f}^{k-1},g\right)\right],$$

Where $\left(ad_{f}^{k},\;g\right)$ is the adjoint operator and the superscripts the adjoint operator and the superscripts represent the order of the Lie bracket. With formal definitions of the input function Eq. (2) and its higher Lie brackets Eq. (14) from 1 to *n*, where *n* is the order of the system matrix *A*_NL_(**x**(*t*)), the nonlinear controllability matrix is defined as

$$\begin{array}{l} Q\equiv \left[g\text{,}\left(ad_{f}^{1},g\right),\ldots ,\left(ad_{f}^{n},g\right)\right]\\ \;\;\;\; \end{array}$$

$$\;=\left[g\text{,}\left[f\text{,}g\right],\left[f\text{,}\left[f\text{,}g\right]\right],\ldots ,\left[f\text{,}\left(ad_{f}^{n-1},g\right)\right]\right].$$

### D. Observability and controllability indices

In systems with real numbers, calculation of the Kalman rank condition may not yield an accurate measure of the relative closeness to singularity (conditioning) of the observability matrix. It was demonstrated in Ref. [26] that the calculation of a matrix condition number [36] would provide a more robust determination of the ill conditioning inherent in a given observability matrix, since condition number is independent of scaling and is a continuous function of system parameters (and states in the generic nonlinear case). We use the inverted form of the observability index *δ*(**x**) given in Ref. [26] so that 0 ≤*δ*(**x**) ≤ 1,

$$\delta \left(x\right)=\frac{\left|\sigma_{\min}\left[O^{T}O\right]\right|}{\left|\sigma_{\max}\left[{}^{T}O\right]\right|},$$

where σ_min_ and σ_max_ are the minimum and maximum singular values of *O*^T^*O*, respectively, and *δ*(**x**) =1 indicates full observability while *δ*(**x**) = 0 indicates no observability [37]. Similarly, the controllability index is just Eq. (16) with the substitution of *Q* for *O.*

## III. OBSERVABILITY AND CONTROLLABILTY OF 3-NODE FITZHUGH-NAGUMO NETWORK MOTIFS

### A. Fitzhugh-Nagumo system dynamics

The Fitzhugh-Nagumo (FN) equations [38,39] comprise a general representation of excitable neuronal membrane. The model is a two-dimensional analog of the well-known Hodgkin-Huxley model [40] of an axonal excitable membrane. The nonlinear FN model can exhibit a variety of dynamical modes, which include active transients, limit cycles, relaxation oscillations with multiple time scales, and chaos [38,41]. A nonlinear connection function will be used to emulate properties of neuronal synapses.

The system dynamics at a node are given by the (local second-order) state space

$${\dot{\upsilon }}_{i}=c\left(\upsilon_{i}-\frac{\upsilon_{i}^{3}}{3}-w_{i}+\sum f_{NL}(\upsilon_{j},d_{ij})+I\right),\;$$

$${\dot{w}}_{i}=\upsilon_{i}-bw_{i}+a,$$

where *i =* 1,2,3 for the 3-node system, *v*_*i*_ represents membrane voltage of node i, *w*_i_ is recovery, *d*_ij_ is the internodal distance from node *j* to *i*, *v*_j_ is the voltage of neighbor nodes with *j=1,2,3* and *j* ≠ *i*, input current *I*, and the system parameters defined above in Eqs. (13) and (15), the observability and controllability matrices are a function of the states, which means a dependence on the particular trajectory taken in phase space. In the following analysis, we are interested in directed information flow between nodes as a function of various topological connection motifs, connection strengths, and input forcing functions (which provide different trajectories through phase space). Each motif is representative of a unique combination of directed connections between the three nodes with and without latent symmetries. The nonlinear connection function commonly used in neuronal modeling [42] takes the form of the sigmoidal activation function of neighboring activity (a hyperbolic tangent) and an exponential decay with internodal distance. We utilize various coupling strengths to determine the effects on the observability (controllability) of the network. Our coupling function takes the form

$$f_{\text{NL}}(\upsilon ,d)=\frac{k}{2}\left[\tanh\left(\frac{\upsilon -h}{2m}\right)+1\right]e^{-d}$$

The sigmoid parameters *k*=1, *h*=0, *m* = 1/4 are set such that *f*_NL_(*v*,*d*) has an ouput range [0,1] for the input interval [–2, 2], Which is the range of the typical FN voltage variable. To introduce heterogeneity for symmetry breaking a 10% variance noise term is added to each of the *d*_ij_ terms (there are six total possible coupling terms *d*_12_; *d*_13_;…, etc.).

In this configuration, inputs from neighboring nodes act in an excitatory-only manner, while the driving input current was a square wave I=0.25$\left[\sum_{n=-\infty }^{\infty }\Pi \left(\omega t-nT\right)+1\right]$ (where ⊓ is the rectangular function, *ω* = 2*π*/5, and *T* = 16$\frac{2}{3}$ ) applied to all three nodes to provide a limit cycle regime to the network; for the limit-cycle regime generated in the original paper by Fitzhugh [38], the driving current input was constant *I* = −0.45 (with the system parameters mentioned above), which we also explore. Chaotic dynamics were generated with a slightly different square wave input [41] $I=0.1225\left[\sum_{n=-\infty }^{\infty }⊓(\omega t-nT)+1\right]$ (with ω = 2π/1.23 and *T* = 2.7891) also applied to all three nodes. These various driving input regimes allow a wider exploration of the phase space of the system as each driving input commands a different trajectory, which will in turn influence the observability and controllability matrices.

### B. Network motifs and simulated data

**FIG. 1.**
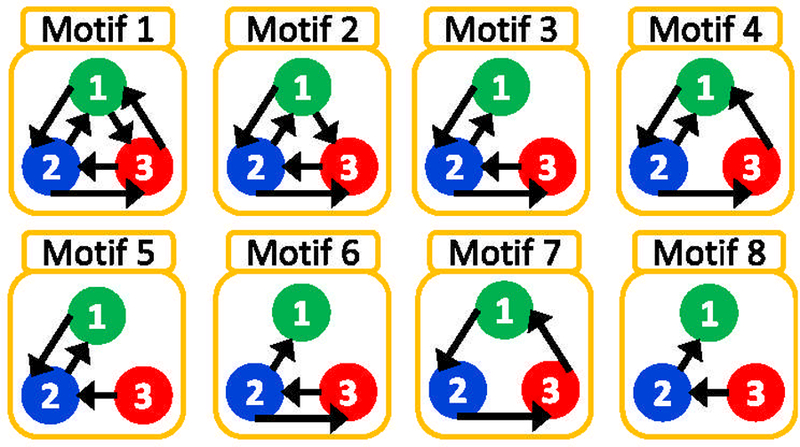
The eight different 3-node network connection motifs studied.

**FIG. 2.**
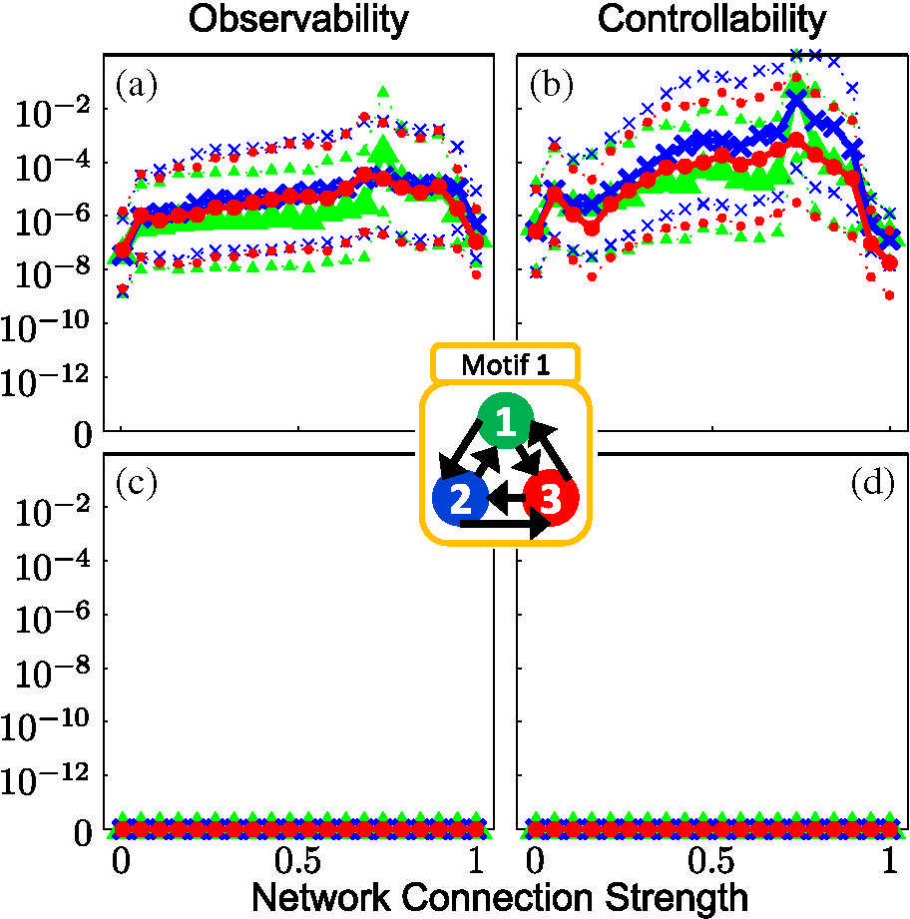
Calculation of (a),(c) observability and (b),(d) controllability indices for motif 1 for a chaotic dynamical regime, as measured from each node (green triangles, 1; blue crosses, 2; red dots, 3). The thick lines and symbols mark the mean values of each distribution of indices for each coupling strength, while the smaller symbols and dotted lines represent the 1 standard deviation confidence intervals. Plots in the top row represent the results computed with symmetry-breaking heterogeneous couplings while plots in the bottom row are those with identical coupling strengths.

**FIG. 3.**
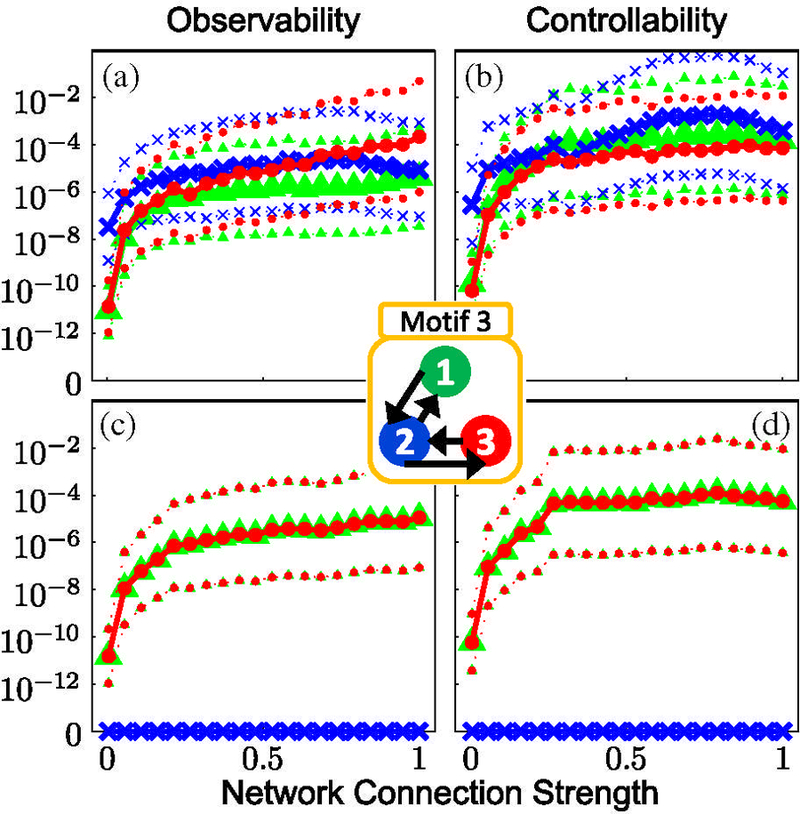
Same as Fig. 2, except calculations are for motif 3. The calculations show that the reflection symmetry in the network topology causes zero observability and controllability for the symmetric case of observing or controlling from node 2 with identical coupling strengths (c),(d).

**FIG. 4.**
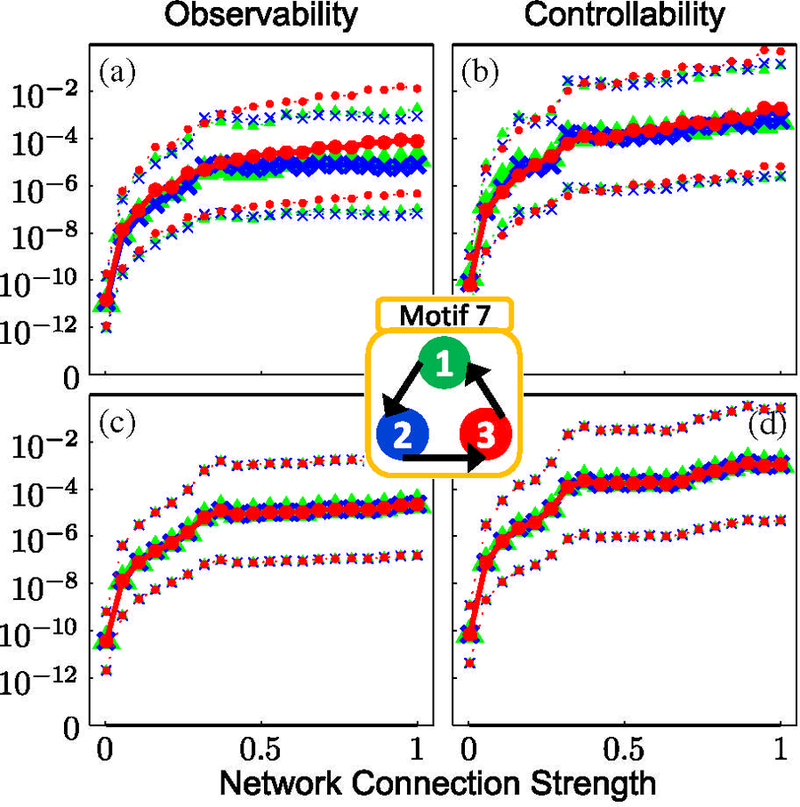
Same as Fig. 2, except calculations are for motif 7. The calculations show that the particular rotational symmetry in the network topology has no ill effect on observability and controllability for the symmetric case of identical coupling strengths (c), (d) as compared to the broken symmetry in panels (a) and (b).

**FIG. 5.**
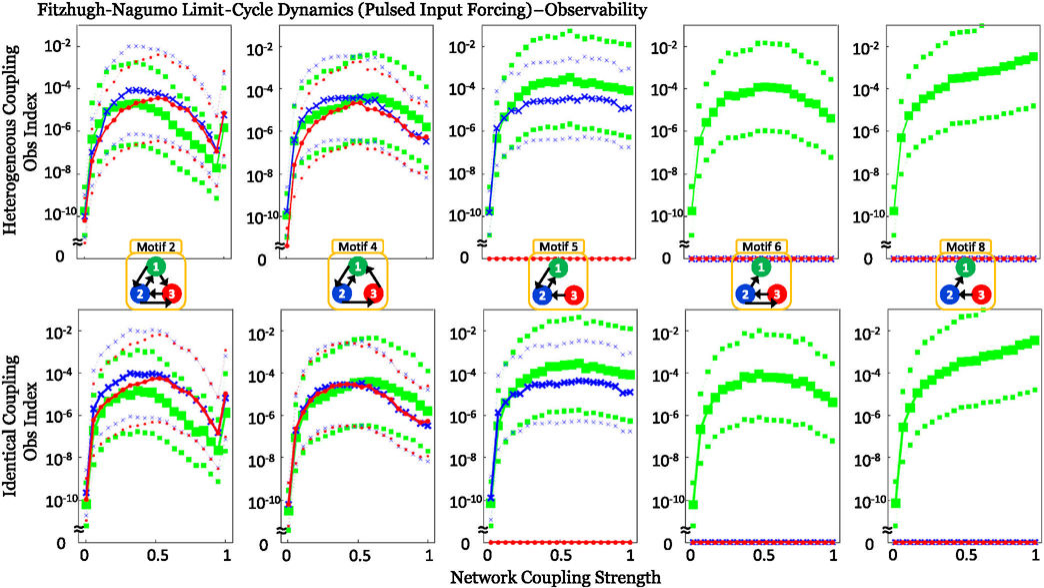
Calculation of observability indices for each of the FN network motifs with no underlying group symmetries for a pulsed input limit-cycle dynamical regime, as measured from each node (green squares, 1; blue crosses, 2; red dots, 3). The thick lines and symbols mark the mean values while the smaller symbols and dotted lines represent the 1 standard deviation confidence intervals. Plots in the top row are computed with heterogeneous couplings while identical coupling strengths are in the bottom row. The calculations show the effect of network coupling strength on observability; motifs 5, 6, and 8 show no observability from node 3 in motif 5, and from nodes 2 and 3 in motifs 6 and 8 due to structural isolation.

**FIG. 6.**
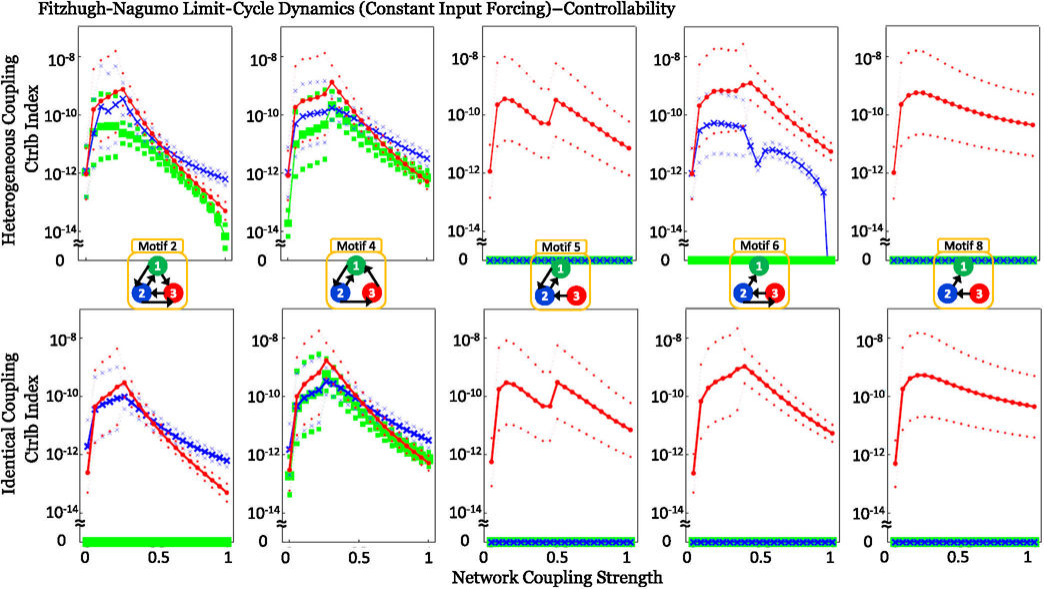
Calculation of controllability indices for each of the FN network motifs with no underlying group symmetries for a limit-cycle dynamical regime with constant input current I = −0.45; all other details are the same as in Fig. 5. In particular, notice that local input-output symmetries cause zero controllability when controlling motif 2 from node 1 or motif 6 from node 2.

As we are interested in the effect of connection topology on observability and controllability, we study the simplest nontrivial network: a 3-node network. Such small network motifs are highly overrepresented in neuronal networks [43,44]. For each network motif shown in Fig. 1, we compute the observability (controllability) indices for various measurement nodes, connection strengths, and driving inputs (dynamic regimes). Measurements of v_i_ for each motif are from each one of the nodes *i* = 1, 2, or 3. Simulated network data are used to compute the observability (controllability) index for two cases: (1) where the system parameters for all three nodes and connections are identical, and (2) where the nodes have a heterogeneous (10% variance) symmetry-breaking set of coupling parameters. To create simulated data, the full six-dimensional FN network equations are integrated from the same initial conditions with the same driving inputs for each node via a Runge-Kutta fourth-order method with time step Δ*t* = 0.04 for 12 000 time steps (with the initial transient discarded) in MATLAB for each test case: (1) limit-cycle and (2) chaotic dynamical regimes, with (a) identical and (b) heterogeneous coupling (the nodal parameters remain identical throughout). Convergence of solutions is achieved when Δ*t* is decreased to 0.04. Data are then imported into Mathematica and inserted into symbolic observability and controllability matrices (computed for each node), which are then numerically computed to obtain the observability (controllability) indices for each coupling strength. The indices are then averaged over the integration paths starting from random initial conditions. These calculations are summarized in Figs. 2–6 for observability and controllability, in the chaotic, pulsed limit-cycle, and constant input limit-cycle dynamical regimes. To facilitate others replicating our work, we have archived extensive code in MATLAB and *Mathematica* in the Supplemental Material [45].

## IV. RESULTS

### A. Motifs with symmetry

For motif 1, the data show that a system with full **S**_**3**_ symmetry (due to the connection topology and identical nodal and coupling parameters) generates zero observability (controllability) over the entire range of coupling strengths [Figs. 2(c) and 2(d)]. Similarly, no observability (controllability) is seen from node 2 in motif 3, which has a reflection **S**_**2**_ symmetry across the plane through node 2 [Figs. 3(c) and 3(d)]. Interestingly, the cyclic symmetry of motif 7 does not cause loss of observability (controllability) as shown in Fig. 4; motif 7 has rotational **C**_**3**_ symmetry and valance 1 connectivity (1 input, 1 output). In motifs 1 and 3 the effect of the symmetry is partially broken by introducing a variation in the coupling terms, and the results show nonzero observability (controllability) indices in the plots for such heterogeneous coupling [plots (a) and (b) in Figs. 2 and 3] with a dependence on the coupling strength.

**FIG.7.**
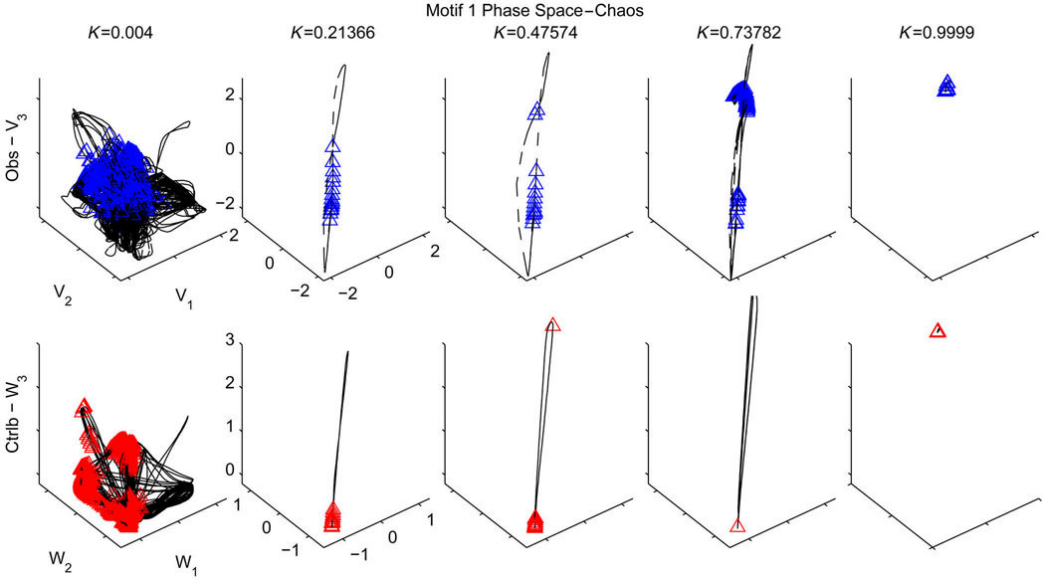
The three dimensional phase space for v and w, showing trajectories in motif 1 as measured from node 1 for arrange of connection strengths(weak to strong heterogeneous coupling *K*,from left to right,respectively).In the first row,blue triangles mark locations in phase space where observability is higher than the mean for the trajectory, while the second row contains a phase space trajectory for *w* and red triangles mark the higher than average controllability. The broken symmetry of the heterogeneous network has trajectories that visit locations in the phase space that vary widely in observability and controllability with a log-normal distribution (see the Appendix).

Of particular interest is the substantial loss of observability (controllability) as the coupling strengths increase to critical levels for systems containing latent structural symmetries in the presence of heterogeneity [motifs 1 and 3, plots (a) and (b) in Figs. 2 and 3]. That is, increasing the coupling strengths when recording (stimulating) from any node in motif 1 or node 2 in motif 3 degrades observability (controllability) as coupling strength increases. A study of the 3D phase plots of the FN voltage variable in motif 1 (as a function of coupling strength for chaotic dynamics) reveals a blowout bifurcation [46] at lower values of coupling strengths (Fig. 7), and at higher levels, generalized synchrony [47] and increased observability (controllability), and finally the subsequent decrease in observability (controllability) at the highest levels of coupling strength [motif 1 as observed (controlled) from any node in Fig. 2]. This is demonstrated in motif 1 (Fig. 7), where a bifurcation in the dynamics causes the wandering trajectories at weak coupling strengths to collapse onto the limit-cycle attractor at stronger coupling strengths, and at the strongest coupling the dynamics reveal a reverse Hopf bifurcation from the limit cycle back into a stable equilibrium.

Although motif 7 contains symmetry, the observability and controllability measures appear unaffected by the presence of this symmetry; further insight into why this happens in such networks requires group representation theory and is presented in Sec. V.

### B. Motifs without symmetry

Local output symmetries occur in motifs 2 and 6 when controlling from the first and second node, respectively (green and blue traces in Fig. 6), which is remedied by the disambiguating effect of parameter variation. Additionally, as in the motifs with symmetry, the broken local symmetries lose controllability as coupling strength further increases, evident in motifs 2 and 6 in Fig. 6. In the cases where the indices are zero without symmetries (motifs 5, 6, and 8 in Figs. 5 and 6), the motif must contain one or more structurally isolated nodes and, hence, are not structurally controllable or observable. From the viewpoint of observability, this means that information from the isolated node (s) cannot reach the measured node as the two are not connected in that direction [10,12]; for controllability, this means that the isolated node(s) is not reached by the controlled node due to the two not being connected in that direction [7]. This structural nodal isolation is exemplified in motif 8 (in Figs. 5 and 6), where the network is only observable from node 1, and only controllable from node 3.

Additionally, the plots in Figs. 5 and 6 show counterintuitively that as coupling strength increases, the observability (controllability) indices can increase to an optimal value, and then begin to decrease as coupling strength increases past this critical coupling value.

## V. SYMMETRIC NETWORK OBSERVABILITY AND CONTROLLABILITY VIA GROUP REPRESENTATION THEORY

For linear time-varying systems, Rubin and Meadows [17] used the theory of group representations [16,19,20,48] to show how a (circuit) network containing group symmetries would be noncontrollable or nonobservable due to symmetries (termed NCS or NOS, respectively). The analysis involves first determining the irreducible representations of the symmetry group of the system equations, then constructing an orthogonal basis (called a symmetry basis) from the irreducible representations which transforms the system matrix A(*t*) into block diagonal form (also called modal form). Inspection of the fully transformed system from Eq. (2) reveals if the NCS or NOS property is present via zeros in a critical location of decoupled block diagonal decomposition *Â*; *B̂*; *Ĉ*, i.e., the form

$$\frac{d}{dt}\left[\begin{array}{c} Z_{1}\\ Z_{2} \end{array}\right]=\underset{\overset{\wedge }{\text{A}}}{\underset{︸}{\left[\begin{array}{cc} A_{1} & 0\\ 0 & A_{2} \end{array}\right]}}\left[\begin{array}{c} Z_{1}\\ Z_{2} \end{array}\right]+\underset{\overset{\wedge }{B}}{\underset{︸}{\left[\begin{array}{c} B_{1}\\ 0 \end{array}\right]}}u(t),\;$$

$$y(t)=\underset{\overset{\wedge }{C}}{\underset{︸}{[\begin{array}{cc} C_{1} & 0] \end{array}}}\left[\begin{array}{c} Z1\\ Z2 \end{array}\right]$$

where the transformed system Eq. (19) in partitioned form above is noncontrollable and nonobservable (not completely controllable or observable). This can be seen by inspection, as the zeros present in the partitioned measurement and control functions *Ĉ* and *B̂* leave the transformed system unable to measure or control the mode associated with *Z*_2_ as neither *u*(*t*) or *Z*_1_ is present in the equation for *Z*_2_, and *Z*_2_ does not appear in the output. In the next section, we summarize the minimum background components of groups and representations (without proofs) in order to further gain insight into how symmetry effects the controllability and observability of our networks.

### A. Symmetric groups and representations

A symmetry operation on a network is a permutation (in this case nodes) that results in exactly the same configuration as before the transformation was applied. The symmetric group **S**_**n**_ consists of all permutations on n symbols, called the order of the group *g* = *n*!. The shorthand method of denoting a permutation operation R of nodes in a network is written (123), where node 1 is replaced by node 2 and node 2 by node 3. This is called a cycle of the permutation [16], and with it we can define all of the permutations of **S**_**n**_. Three of the network motifs we study here contain topological symmetries (Figs. 2–4); motif 1 has **S**_**3**_ symmetry, motif 3 has **S**_**2**_ symmetry, and motif 7 contains **C**_**3**_ symmetry (see Ref. [20] for a rigorous classification of various forms of symmetry), and each of these groups comprise the following sets of permutation operations *R*:

$$\text{R}:\;S_{\text{3}}=\left\{E,\sigma 1,\sigma 2,\sigma 3,C3,C_{3}^{2}\right\}\;\;\;\;\;\;$$

**FIG. 8.**
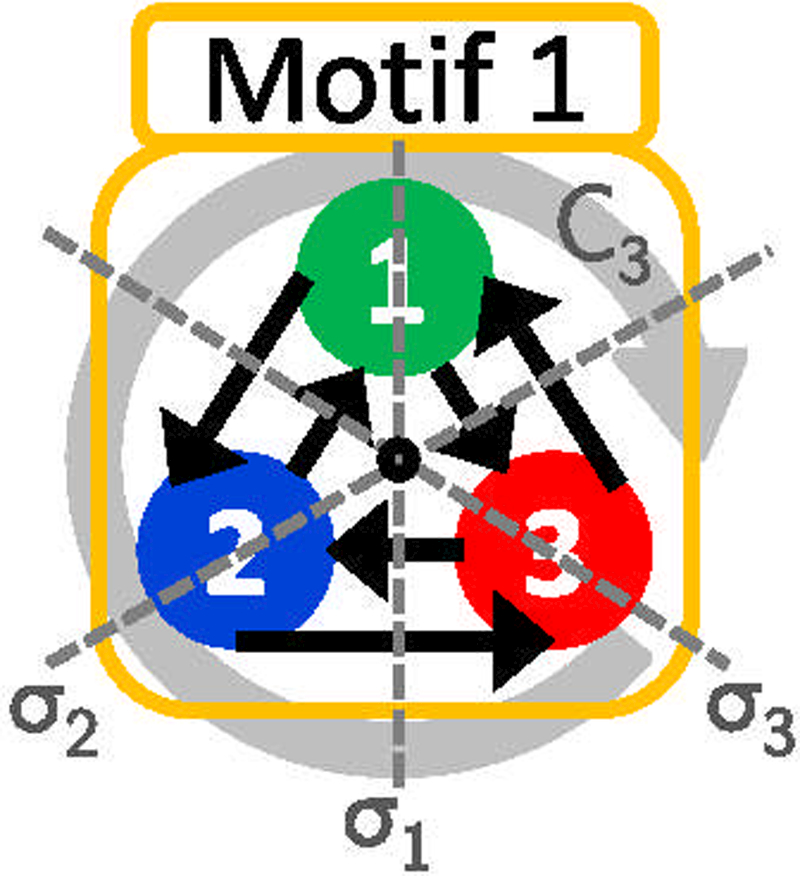
Graphic illustration of symmetry axes σ_*n*_ with *n* = 1;2;3 and the cyclic rotation symmetry *C*_3_ about an axis perpendicular to the plane of the page.

 = {E=(1)(2)(3)

$$\sigma_{1}=(23),\sigma_{2=}(13),\sigma_{1}=(12)\;$$

$$C_{3=}(132),C_{3}^{2}=(123)\},$$

where *E* is the identity operation, *σ*_*n*_ is a reflection across the nth axis in Fig. 8, and C_3_ and $C_{3}^{2}$ are two cyclic rotations where *C*_*n*_ denotes a rotation of the system by *2π/n* rad where the system remains invariant after rotation [20]. **S**_**2**_ and **C**_**3**_ symmetry in motifs 3 and 7, respectively, are subgroups of **S**_**3**_:

$$S_{2}=\{E,\sigma_{2}\},$$

$$C_{3}=\{E,C3,C_{3}^{2}\}.$$

The permutation operations *R* in these symmetric groups can also be represented by monomial matrices [49] *D*(*R*): where *D*(*R*) in Eq. (22) is a three-dimensional representation of **S**_**3**_ group symmetry (for our three node motifs); a representation *D*(*R*) for **S**_**2**_ and **C**_**3**_ group symmetry are just the matrices above in Eq. (22) corresponding to the sets of group elements given in Eq. (21).

$$\underset{E}{\left[\begin{array}{ccc} 1 & 0 & 0\\ 0 & 1 & 0\\ 0 & 0 & 1 \end{array}\right]}\underset{\sigma_{1}}{\;\left[\begin{array}{ccc} 1 & 0 & 0\\ 0 & 0 & 1\\ 0 & 1 & 0 \end{array}\right]}\;\;\underset{\sigma_{2}}{\left[\begin{array}{ccc} 1 & 0 & 0\\ 0 & 1 & 0\\ 0 & 0 & 1 \end{array}\right]\;}\underset{\sigma_{3}}{\left[\begin{array}{ccc} 0 & 1 & 0\\ 0 & 0 & 1\\ 1 & 0 & 0 \end{array}\right]}\underset{C_{3}}{\left[\begin{array}{ccc} 0 & 1 & 0\\ 0 & 0 & 1\\ 1 & 0 & 0 \end{array}\right]}\underset{C_{3}^{2}}{\;\left[\begin{array}{ccc} 0 & 0 & 1\\ 1 & 0 & 0\\ 0 & 1 & 0 \end{array}\right]}$$

A group of matrices *D*(·) is said to form a representation of a group **S**_**n**_ if a correspondence (denoted ~) exists between the matrices and the group elements such that products correspond to products; i.e., if R_1_ ~ *D*(*R*_1_) and *R*_2_ ~ *D*(*R*_2_), then the composition (*R*_1_*R*_2_)~*D*(*R*_1_)*D*(*R*_2_) = *D*(*R*_1_*R*_2_) (Definition 12 in Ref. [17]); this is known as a homomorphism of the group to be represented, and if the correspondence is one to one, the representation is isomorphic and called a “faithful” representation of the group

Theorem 2 from Ref. [17] establishes the connection between group theory and the linear network system equations (2), by demonstrating that the monomial representation *D*(*R*) of symmetry operations *R* is conjugate (commutes) with the network system matrix *A* in Eq. (2):

$$D^{-1}(R)A(t)D(R)=A(t),\;\;\forall R\in S_{n}$$

where *D*(*R*) shows how the states of the system equations transform under the symmetry operation *R* and form a reducible representation [16,50] of the symmetric group **Sn**. A representation is said to be reducible if it can be transformed into a block-diagonal form via a similarity transformation α, and irreducible if it is already in diagonal form ; a reducible representation *D*(*R*) that has been reduced to block-diagonal form $\hat{D}(R)$ will have *k* nonzero submatrices along the diagonal that define the irreducible representations *D*^(*p*)^(*R*), *p* = 1,…,*k* of the group **S**_**n**_[17],

$$a^{†}D(R)\alpha =\overset{\wedge }{D}(R),\;\;\forall R\in S_{n},\;$$

$$\overset{\wedge }{D}(R)=\left[\begin{array}{ccc} D_{l_{1}}^{\left(1\right)} & & 0\\ & \ddots & \\ 0 & & D_{l_{k}}^{\left(k\right)} \end{array}\right]$$

where † represents the complex conjugate transpose of *α*, *l*_p_ is the dimension of *D*^(*p*)^(*R*), and the number of irreducible representations *k* equals the number of classes the group elements *R* are partitioned into. This can be found by computing the trace of each representation in *D*(*R*), ∀*R*—called the character of the representation—and collecting those that have the same trace into separate classes Cp, *p* = 1,…, *k*, which define sets of conjugate elements [20]. The character of *D*(*R*) is defined as

$$\chi (R)=Tr[D(R)],\;\;\;\forall R\in S_{n}.$$

The key to forming irreducible representations in Eq. (4) is that the transform *α* needs to reduce each representation matrix *D*(*R*) to diagonal form for every group element *R* in **S**_**n**_. In Eq. (24), the dimension of each irreducible representation *l*_p_ can be found from the fact that the irreducible representations of the group form an orthogonal basis in the *g*-dimensional space of the group, and since there can be no more than *g* independent vectors in the orthogonal basis, it can be shown [48] that

$$\sum_{p=1}^{k}l_{p}^{2}=g$$

where the sum is over the number of irreducible representations (or classes of conjugate group elements) *k*. Some of the irreducible representations where the sum is over the number of irreducible representations sentations (or classes of conjugate group elements) *k*. Some of the irreducible representations *D*^(*p*)^(*R*) will appear in $\hat{D}(R)$ more than once while others may not appear at all; the character of the representation completely determines this, and the number of times *a*_*p*_ that D^(p)^(R) appears in$\hat{D}(R)$ is defined in Ref. [20]

$$a_{p}=\frac{1}{g}\sum_{R}x^{\left(p\right)}(R)*\chi (R)$$

as where *χ*^(p)^(R) is the trace of *D*^(*p*)^(R), the asterisk denotes complex conjugate, and *χ*(*R*) is the trace of *D*(*R*).

### B. Construction of the similarity transform α

We examine motif 3 in Fig. 3, which has **S**_**2**_ symmetry [51]. Determined from Eq. (5), there are two classes of group elements *C*_1_ = {*E*} and C_2_ = {σ_2_}, and reduction of D(R) yields the two, one-dimensinal [*l*_1_=*l*_*2*_=1 computed from Eq. (26)] irreducible representations *D*^(1)^(*R*) and D^(2)^(R) of **S2:**

$$\begin{array}{ccc} R & E & \sigma_{2}\\ D^{\left(1\right)}(R) & 1 & 1\\ D^{\left(2\right)}(R) & 1 & -1 \end{array}$$

where each entry in *D*^(*p*)^ corresponds to the elements of *D*(*R*) above in Eq. (22), where *R* = {*E*, σ_2_} as in Eq. (2^1^), and from Eq. (27), *D*^(1)^ (*R*) appears two times while *D*^(2)^ (*R*) appears once in *D*(*R*).

A procedure for transforming the reducible representation *D*(*R*) of a symmetry group S_**n**_ to block-diagonal form is presented in Refs [17,50]. A unitary transformation α is constructed from the normalized linearly independent columns of the *n* × *n* generating matrix $G_{i}^{\left(p\right)}$,

$$G_{i}^{\left(p\right)}=\sum_{R}D^{\left(p\right)}{\left(R\right)}_{ii}^{*}D(R)$$

where *D*^(*p*)^(*R*)_ii_ is the (*i*,*i*)th diagonal entry of an *l*_p_dimensional irreducible representation *p* (hence, *i* = 1,…,*l*_p_) of the symmetry group **S**_**n**_ and the asterisk denotes complex conjugate. Each matrix $G_{i}^{\left(p\right)}$ will contribute a_p_ linearly independent columns from Eq. (27) to form the coordinate transformation matrix α. Using Eqs. (28) and (29) and iterating through all *l*_p_ rows of each of the *k* irreducible representations in Eq. (24), we construct *α* for motif 3:

$$G_{1}^{\left(1\right)}=\sum_{R\in S_{2}}D^{\left(1\right)}{\left(R\right)}_{11}^{*}D(R)\;$$

$$=1\left[\begin{array}{ccc} 1 & 0 & 0\\ 0 & 1 & 0\\ 0 & 0 & 1 \end{array}\right]+1\left[\begin{array}{ccc} 0 & 0 & 1\\ 0 & 1 & 0\\ 1 & 0 & 0 \end{array}\right]=\left[\begin{array}{ccc} 1 & 0 & 1\\ 0 & 2 & 0\\ 1 & 0 & 1 \end{array}\right]$$

where each linearly independent column of *G* is a column of *α*. After normalizing, we have

$$\left[\begin{array}{c} 1\\ 0\\ 1 \end{array}\right],\left[\begin{array}{c} 0\\ 2\\ 0 \end{array}\right]\underset{normalize}{\to }\left[\begin{array}{c} \frac{1}{\sqrt{2}}\\ 0\\ \frac{1}{\sqrt{2}} \end{array}\right],\left[\begin{array}{c} 0\\ 1\\ 0 \end{array}\right]=\left[\begin{array}{cc} \alpha_{11} & \alpha_{21}\\ \alpha_{12} & \alpha_{22}\\ \alpha_{13} & \alpha_{23} \end{array}\right]$$

which defines the first and second columns of *α.* Continuing, we have

$$G_{1}^{\left(2\right)}=\sum_{R\in S_{2}}D\left(2\right)(R)*{}_{11}D(R)\;$$

$$=1\left[\begin{array}{ccc} 1 & 0 & 0\\ 0 & 1 & 0\\ 0 & 0 & 1 \end{array}\right]-1\left[\begin{array}{ccc} 0 & 0 & 1\\ 0 & 1 & 0\\ 1 & 0 & 0 \end{array}\right]=\left[\begin{array}{ccc} 1 & 0 & 1\\ 0 & 2 & 0\\ 1 & 0 & 1 \end{array}\right],$$

which yields the final column of *α* (after normalization):

$$\left[\begin{array}{c} 1\\ 0\\ -1 \end{array}\right]\underset{normalize}{\to }\left[\begin{array}{c} \frac{1}{\sqrt{2}}\\ 0\\ -\frac{1}{\sqrt{2}} \end{array}\right]=\left[\begin{array}{c} \alpha_{31}\\ \alpha_{32}\\ \alpha_{33} \end{array}\right]$$

Now, the coordinate transformation matrix *α* is

$$\alpha =\left[\begin{array}{ccc} \frac{1}{\sqrt{2}} & 0 & \frac{1}{\sqrt{2}}\\ 0 & 1 & 0\\ \frac{1}{\sqrt{2}} & 0 & -\frac{1}{\sqrt{2}} \end{array}\right]$$,

Motif 3 in Fig. 3 has connection matrix *A*_3_:

$$A_{3}=\left[\begin{array}{ccc} 0 & 1 & 0\\ 1 & 0 & 1\\ 0 & 1 & 0 \end{array}\right]$$

To control from nodes 1, 2, and 3, respectively, the *B* matrix takes the form

$$B_{1,2,3}=\left[\begin{array}{c} 1\\ 0\\ 0 \end{array}\right],\left[\begin{array}{c} 0\\ 1\\ 0 \end{array}\right],\left[\begin{array}{c} 0\\ 0\\ 1 \end{array}\right],$$

and to observe from nodes 1, 2, and 3, respectively, the *C* matrix takes the form

$$c_{1,2,3}=\left[\begin{array}{ccc} 1 & 0 & 0 \end{array}\right],\left[\begin{array}{ccc} 0 & 1 & 0 \end{array}\right],\left[\begin{array}{ccc} 0 & 0 & 1 \end{array}\right]$$

The block-diagonalized system $\left({\hat{A}}_{3},\hat{B},\hat{C}\right)$ is formed with the substitution Z=*α*^†^*x* and (*A*_3_, *B*, *C*) in Eqs. (35)–(37) becomes

$${\hat{A}}_{3}:\alpha^{{}^{†}}A_{3}\alpha =\left[\begin{array}{ccc} 0 & \sqrt{2} & 0\\ \sqrt{2} & 0 & 0\\ 0 & 0 & 0 \end{array}\right],\;$$

$$\overset{\;\;\;\;\;}{\overset{\wedge }{B}}:\alpha^{{}^{†}}B_{1,2,3}=\left[\begin{array}{c} \frac{1}{\sqrt{2}}\\ 0\\ \frac{1}{\sqrt{2}} \end{array}\right],\left[\begin{array}{c} 0\\ 1\\ 0 \end{array}\right],\left[\begin{array}{c} \frac{1}{\sqrt{2}}\\ 0\\ -\frac{1}{\sqrt{2}} \end{array}\right],\;$$

$$\overset{\;\;\;\;\;}{\overset{\wedge }{C}}:C_{1,2,3}\alpha =\left[\begin{array}{ccc} \frac{1}{\sqrt{2}} & 0 & \frac{1}{\sqrt{2}} \end{array}\right],\left[\begin{array}{ccc} 0 & 1 & 0 \end{array}\right],\left[\begin{array}{ccc} \frac{1}{\sqrt{2}} & 0 & \frac{1}{\sqrt{2}} \end{array}\right]$$

By inspection of the transformed system Eq. (38), it becomes clear that motif 3 is non-controllable and nonobservable from node 2 due to symmetry alone (NCS and NOS); i.e. the transformed system in modal coordinates,

$$\frac{d}{dt}\left[\begin{array}{c} Z_{1}\\ Z_{2}\\ Z_{3} \end{array}\right]=\left[\begin{array}{ccc} 0 & \sqrt{2} & 0\\ \sqrt{2} & 0 & 0\\ 0 & 0 & 0 \end{array}\right]\;\;\left[\begin{array}{c} Z_{1}\\ Z_{2}\\ Z_{3} \end{array}\right]+\left[\begin{array}{c} 0\\ 1\\ 0 \end{array}\right]u\left(t\right),\;$$

$$y\left(t\right)=\left[\begin{array}{ccc} 0 & 1 & 0 \end{array}\right]\left[\begin{array}{c} Z_{1}\\ Z_{2}\\ Z_{3} \end{array}\right],$$

is NCS and NOS as the mode associated with Z3 cannot be reached by the input ${\overset{\;\;\;\;\;}{\hat{B}}}_{2}$nor can its measurement be inferred from the output ${\hat{C}}_{2}$as in Eq. (19)

The procedure to reduce motif 1 is accomplished in a similar fashion (full computation of *α* is detailed in the Appendix) and the connection matrix *A*_1_ and its reduced form *Â*_1_ is

$$A_{1}=\left[\begin{array}{ccc} 0 & 1 & 1\\ 1 & 0 & 1\\ 1 & 1 & 0 \end{array}\right],\;\;\;\;\;\;\;\;\;{Â}_{1}=\left[\begin{array}{ccc} 2 & 0 & 0\\ 0 & -1 & 0\\ 0 & 0 & -1 \end{array}\right],$$

while the transformed B and C matrices in Eqs. (36) and (37) are

$${\hat{B}}_{{}_{1,2,3}}=\left[\begin{array}{c} \frac{1}{\sqrt{3}}\\ \sqrt{\frac{2}{3}}\\ 0 \end{array}\right],\left[\begin{array}{c} \frac{1}{\sqrt{3}}\\ \frac{-1}{\sqrt{6}}\\ \frac{1}{\sqrt{2}} \end{array}\right],\left[\begin{array}{c} \frac{1}{\sqrt{3}}\\ \frac{-1}{\sqrt{6}}\\ \frac{1}{\sqrt{2}} \end{array}\right],\;\;\;\;\;\;\;\;\;\;\;\;\;\;{\hat{C}}_{1,2,3}={\hat{B}}_{1,2,3}^{T}$$

At first glance, it appears that motif 1 is NCS and NOS for measurement and control from node 1 only, and fully controllable and observable from node 2 and 3; however, there is a subtle nuance to the controllability and observability of the diagonal form used in Ref. [17] and consolidated in Eq. (19) to show noncontrollability and nonobservability by inspection.

It is well known that every nonsingular *n* × *n* matrix has *n* eigenvalues *λ*_*n*_, and that a matrix with repeated eigenvalues of algebraic multiplicity *m*_*i*_ will have a degeneracy$1\leq q_{i}\leq m_{i}$ associated with the number of linearly independent eigenvectors for repeated eigenvalue *λ*_*i*_. This degeneracy *q*_*i*_ is also called the geometric multiplicity of *λ*_*i*_, and is equal to the dimension of the null space of $A-I\lambda_{i}$ [52]. When utilizing similarity transforms to reduce a matrix to diagonal (modal) form, this degeneracy in the eigenvectors (brought about by repeated eigenvalues) results in a transformed matrix that is almost diagonal, called the Jordan form matrix. The Jordan form is composed of submatrices of dimension *m*_*i*_—called Jordan blocks—that have ones on the superdiagonal of each Jordan block *J*_*i*_ associated with the generalized eigenvectors of a repeated eigenvalue *λ*_*i*_. The diagonal form in Eq. (19) is a special case of a Jordan form where the matrices on the diagonal are Jordan blocks of dimension one. This is known as the fully degenerate case with *q = mi*, and the Jordan form will have mi separate 1 × 1 Jordan blocks associated with each eigenvalue *λi*.

The observability and controllability of systems in Jordan form hinges on where the zeros appear in the partitioned *C*_*i*_ and *B*_*i*_ matrices, where subscript *i* indicates a partition associated with a particular Jordan block *J*_*i*_. Given in Refs. [52,53], the conditions for controllability and observability of a system in Jordan form are: 1. The first columns of *C*_*i*_ or the last rows of *B*_*i*_ must form a linearly independent set of vectors {*c*_11_…*c*_1*qi*_} or {*b*_1_…*b*_*qie*_} (subscript e indicates the last row) corresponding to the *q*_*i*_ Jordan blocks $J_{1}^{\lambda i}\cdots J_{qi}^{\lambda i}$ for repeated eigenvalue $\lambda_{{}_{i}}$_._ 2.$c_{1p}\neq 0$ or $\;b_{pe}\neq 0$ when there is only one Jordan block $J_{p}^{\lambda i}$ associated with eigenvalue *λ*_*i*_. 3. For single output and single input systems, the partitions of *C*_*i*_ and *B*_*i*_ are scalars—which are never linearly independent—thus, each repeated eigenvalue must have only one Jordan block $J_{i}^{\lambda i}$ associated with it for observability or controllability, respectively. From these criteria, we can now see that the transformed system for motif 1 in Eq. (24) contains three 1 × 1 Jordan blocks, two of which are associated with the repeated eigenvalue *λ*_2_ =−1, which violates condition (3); thus, we conclude it is NCS and NOS.

### C. Motif 7 and networks containing only rotation groups

In Ref. [17], it was shown how the rth component of α vanishes according to the matrices D^(p)^ $\left(R_{r}^{r}\right)$ where $R_{r}^{r}$ represents a subgroup of the group operations (*R*) that transform the *r*th state variable into itself. Subsequently, two theorems were proven that make use of this fact to simplify the analysis of networks that have a single input or output coupled only to the *r*th state variable, which is precisely parallel to our analysis in Sec. IV. A paraphrasing of Theorems 6 and 12 from Ref. [17] for controllability and observability states that such a single input or output network is NCS or NOS if and only if there is an irreducible representation *D*^(*p*)^ (*R*) that appears in *D*(*R*) and

$$\sum_{R_{r}^{r}}S_{r}^{r}D^{\left(p\right)}{\left(R_{r}^{r}\right)}_{ii}^{*}=0$$

for some value of *i*, where$S_{r}^{r}$ is +1 or −1 as $R_{r}^{r}$ transforms state variable *x*_*r*_ into itself with a plus or minus sign [in our motifs, *D*(*R*) is a permutation representation; thus, $S_{r}^{r}$ = +1]. For this theorem to hold, the equality in Eq. (42) must be checked for all possible *p* for *D*^(*p*)^that appear in (*R*) via Eq. (27).

Applying Eq. (42) to motif 7, the irreducible representations for *C*_3_ symmetry are

$$\begin{array}{cccc} R & E & C_{3} & C_{3}^{2}\\ \begin{array}{l} D^{\left(1\right)}(R)\\ D^{\left(2\right)}(R)\\ D^{\left(3\right)}(R) \end{array} & \begin{array}{l} 1\\ 1\\ 1 \end{array} & \begin{array}{l} 1\\ \omega \\ \omega^{2} \end{array} & \begin{array}{l} 1\\ \omega^{2}\\ \omega \end{array} \end{array}$$

where *ω* = *e*^2πi=3^. From the subset Eq. (21) of Eq. (22), we find that the only operation $R_{r}^{r}$ that leaves either node 1, 2, or 3 (state variables *x*_1_, *x*_2_, or *x*_3_) invariant is just the identity operation *E*, and it is straightforward to see that Eq. (42) is not equal to 0 for all choices of *p*, *i*, and r since there is only one group operation that leaves the rth state variable invariant, $R_{r}^{r}$ = *E*, for *r* = 1;2;3. Thus, motif 7 cannot be NCS or NOS and must be controllable and observable from any node. Corollary 1 to Theorem 6 from Ref. [17] contains and expands this result directly to any network with only rotational symmetry (i.e., *C*_*n*_ groups), with the caveat that a network with a state variable that is invariant under all the group operations (motif 7 does not have such a state variable) will be NCS and NOS if the input and output are coupled to that variable.

These representation group theoretic results explain our nonlinear results in Sec. IV, and clearly demonstrate that different types of symmetry have different effects on the controllability and observability of the networks containing them. While we explicitly assume system matrices with zeros on the diagonal (for simplicity of the calculations), these results hold with generic entries on the diagonal as long as those entries are chosen to preserve the symmetry (e.g., the system matrix A for motif 1 and 7 has *a*_11_ = *a*_22_ *a*_33_ and motif 3 has *a*_11_ = *a*_33_, not shown). Linearization of the system equations in Eq. (17) would result in a system matrix *A* with a nonzero diagonal [9], and is typically done in the analysis of nonlinear networks [18] when utilizing such linear analysis techniques. Our computational results demonstrate the utility of this approach in providing insight into the controllability and observability of complex nonlinear networks that have not been linearized.

### D. Application to structural controllability and observability

It is interesting to note that the demonstration of our results above and those in Ref. [17] complement and expand Lin’s seminal theorems on structural controllability [7]. Essentially, a network with system matrix *A* and input function *B* [the pair (*A*, *B*)] are assumed to have two types of entries, nonzero generic entries and fixed entries which are zero. The position of the zero entries leads to the notion of the structure of the system, where different systems with zeros in the same locations are considered structurally equivalent. With this definition of structure, we arrive at the definition for structural controllability, which states that a pair (*A*’, *B*’) is structurally controllable if and only if there exists a controllable pair (*A*”, *B*”)with the same structure as (*A*’, *B*’) The major assumption of this work is that a system deemed to be structurally controllable could indeed be uncontrollable due to the specific entries in *A* and *B*, which for a practical application are assumed to be uncertain estimates of the system parameters and thus subject to modification. While Lin’s theorems did not explicitly cover symmetry, any network pair (*A*, *B*)containing symmetry implies constraints on the nonzero entries in (*A*, *B*), which is necessary to guarantee that symmetry is present. Thus, considering only Ref. [7], a network with symmetry could be structurally controllable (observable [10]) as long as the graph of the system contains no dilations (defined in the Appendix) or isolated nodes, but NCS (NOS) due to the symmetry. These two theorems together paint a more complete picture of controllability (observability) than either alone, as shown in Secs. IV and V, where both are used in concert to explain and understand why certain network motifs are not controllable or observable from particular nodes. Structural controllability (observability) is a more general result, as it does not depend on the explicit nonzero entries of the system pair (*A*, *B*) (necessary, but not sufficient), while a network that has the NCS (NOS) property is due to specific sets of the nonzero entries in (*A*, *B*)that define the symmetry contained by the system.

Additionally, Ref. [7] defined two structures called a “stem” (our motif 8 controlled from node 3) and a “bud” (our motif 7 controlled from any node), which are always structurally controllable. While both are easily shown to be structurally controllable [7], including Theorem 6 and its Corollary 1 from Ref. [17], we can take this a step further and declare that any “bud” network (of arbitrary size) containing only rotations is not only structurally controllable, but also fully controllable (or never NCS). The dual of these structures for observability is also defined in Ref. [10], and Theorem 12 and its Corollary 1 from Ref. [17] completes the statement in a similar fashion for observability. Since networks containing only rotation groups or “buds” in Lin’s terminology are always controllable, we see that in some cases symmetries alone will not destroy the controllability of structurally controllable networks.

## VI. DISCUSSION

Despite the growing importance of exploring observability and controllability in complex graph-directed networks, there has been little exploration of nonlinear networks with explicit symmetries. We here report, to our knowledge, the first exploration of symmetries in nonlinear networks, and show that observability and controllability are a function of the specific type of symmetry, the spatial location of nodes sampled or controlled, the strength of the coupling, and the time evolution of the system.

In networks with structural symmetries, group representation theory provides deep insights into how the specific set of symmetry operations possessed by a network will influence its observability and controllability and can aid in controller or observer design by obtaining a modal decomposition of the network equations into decoupled controllable and uncontrollable (observable and unobservable) subspaces. This knowledge will permit the intelligent placement of the minimum number of sensors and actuators that render a system containing symmetry fully controllable and observable. Additionally, breaking symmetry through randomly altering the coupling strengths establishes substantial observability or controllability that was absent in the fully symmetric case. In cases where increasing the overall level of coupling strength decreases the observability (controllability), such strong coupling eventually pushes the system towards or through a reverse Hopf bifurcation from limit cycle to a stable equilibrium point, where the lack of dynamic movement of the system then severely decreases the observability (controllability). Intuitively, this results from the Lie derivatives (brackets) becoming small as the rate of change of the system trajectories goes to zero. The sensitivity of observability and controllability to the trajectories taken through phase space implies that the choice of control input to a system has to be selected carefully, as a poor choice could drive the system into a region that has little to no controllability or observability, thereby thwarting further control effort and/ or causing observation of the full system to be lost or limited. Furthermore, when using an observer model for observation or control, the regions of local high observability could be utilized to optimize the coupling of the model to a real system by only estimating the full system state when the system transverses observable regions of phase space.

Observation (control) in motifs 2, 3, 4, 5, and 6 suggests a relationship between the degree of connections into and out of a node and its effective observability (controllability). In general, the more direct connections into an observed node, the higher the observability from that node, and the duality suggests that the more direct number of outgoing connections from a controlled node leads to higher controllability than from other less connected nodes. The high degree “hub” nodes were not the most effective driver nodes in complex networks using linear theory [8], and extending nonlinear results to more complex networks with symmetries is a challenge for future work, which may benefit from linear analysis of the connection topology utilizing group representation theory.

When observing kinematics and dynamics of rigid body mechanics obeying Newton’s laws with SE(3) group symmetry, such symmetries must be preserved in constructing an observer (controller) [54]. In the observation of graph-directed networks containing transitive networks, one can observe from any point equivalently within such transitive components [11]. In the control of graph-directed networks, the minimum number of control points is related to the maximal matching nodes [8]. In Ref. [55], contraction theory was used to determine symmetric synchronous subspaces—these spaces actually correspond to our regions without observability or controllability. In fact, the proof of observability is that initial conditions and trajectories do not contract [12]. Furthermore, it is clear that the groupoid input equivalence classes (such as our motifs 6 and 7, see Fig. 21 in Ref. [56]) are not equivalently observable or controllable—note that only one node can serve as an observer node in motif 6 regardless of coupling strength (see Fig. 5). Indeed, whether virtual networks [55] with particular groupoid equivalent symmetries serve as detectors of observability and controllability remains unresolved at this time.

Our deep knowledge of symmetries and observers in classical mechanics [54] does not readily translate to graph-directed networks. No real-world network has exact symmetries. Our topologically symmetric systems with identical components are extreme cases, yet their study reveals important differences in which types of symmetries are or are not observable and controllable. Furthermore, in nonlinear systems, the quality of the mappings from system to observer is the key to estimating the degree of observability or controllability, and our methods can give us insight for any network. Further development of a theory of observability and controllability for nonlinear networks with symmetries is a vital open problem for future work.

## Supplementary Material

Code Archive

## Figures and Tables

**FIG. 9.**
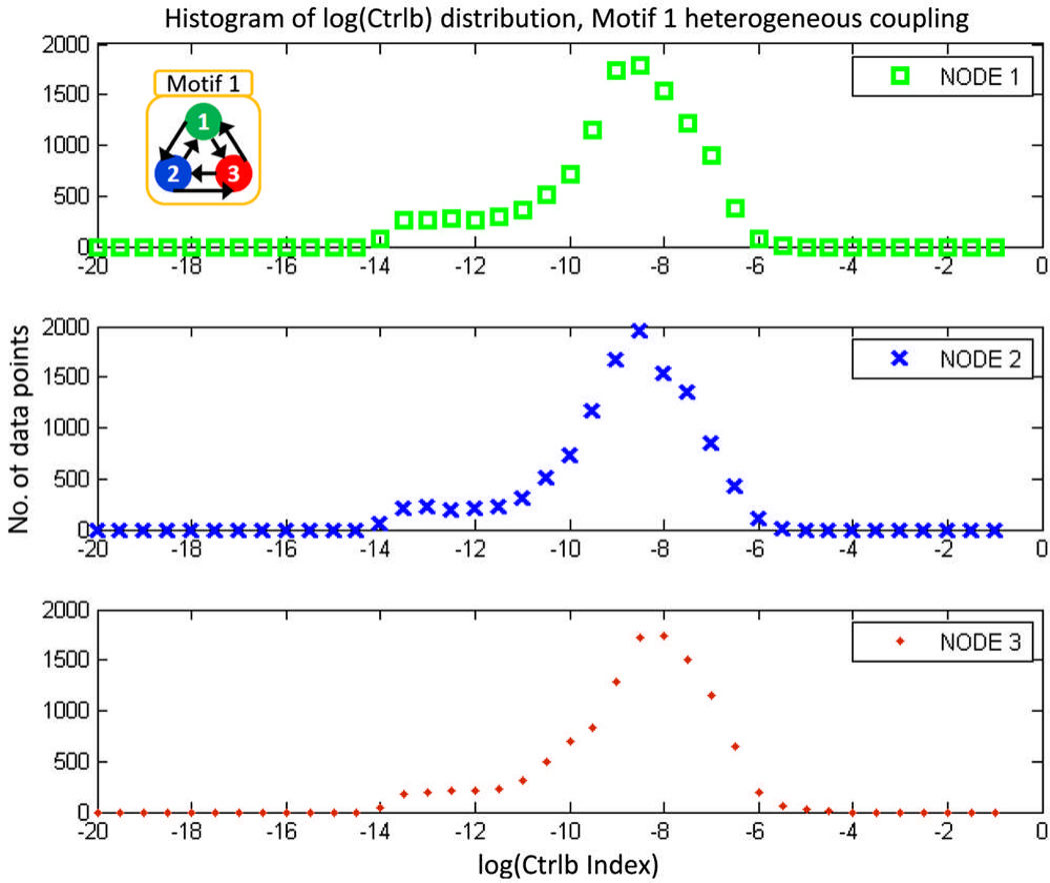
Histogram of the log-scaled controllability indices for motif 1 with heterogeneous coupling and chaotic dynamics.

## APPENDIX

1. Construction of differential embedding map and Lie brackets

As an example case, we begin constructing the observability matrix for motif 1 (shown in Fig. 2), where the Fitzhugh-Nagmuo (FN) network equations form the nonlinear vector field **f**:

$$f\left\{ \begin{array}{l} f_{1}=c\left[\upsilon_{1}-\frac{\upsilon_{1}^{3}}{3}-w_{1}+\sum_{j=2,3}f_{NL}(\upsilon_{j},d_{1j})\right]\\ f_{2}=\upsilon_{1}-bw_{1}+a\\ f_{3}=c\left[\upsilon_{2}-\frac{\upsilon_{2}^{3}}{3}-w_{2}+\sum_{j=1,3}f_{NL}(\upsilon_{j},d_{2j})\right]\\ f_{4}=\upsilon_{2}-bw_{2}+a\\ f_{5}=c\left[\upsilon_{3}-\frac{\upsilon_{3}^{3}}{3}-w_{3}+\sum_{j=1,2}f_{NL}(\upsilon j,d_{3j})\right]\\ f_{6}=\upsilon_{3}-bw_{3}+a \end{array}\right.$$

and the measurement function for node 1 in motif 1 is *y* = C**x**(*t*)=[1,0,0,0,0,0]**x**(*t*)=$\upsilon_{1}$ We construct the differential embedding map by taking the Lie derivatives Eq. (5) from $L_{f}^{0}(y)\;to\;L_{f}^{5}(y)$ as

$$\phi \left\{ \begin{array}{l} \phi_{1}=y=\upsilon_{1}\\ \phi_{2}=\frac{\partial y}{\partial \upsilon_{1}}\cdot f_{1}=f_{1}\\ \phi_{3}=\frac{\partial \phi_{2}}{\partial \upsilon_{1}}f_{1}+\frac{\partial \phi_{2}}{\partial w_{1}}f_{2}+\frac{\partial \phi_{2}}{\partial \upsilon_{2}}f_{3}+\cdots +\frac{\partial \phi_{2}}{\partial w_{3}}f_{6}\\ \phi_{4}=\frac{\partial \phi_{3}}{\partial \upsilon_{1}}f_{1}+\frac{\partial \phi_{3}}{\partial w_{1}}f_{2}+\frac{\partial \phi_{3}}{\partial \upsilon_{2}}f_{3}+\cdots +\frac{\partial \phi_{3}}{\partial w_{3}}f_{6}\\ \phi_{5}=\frac{\partial \phi_{4}}{\partial \upsilon_{1}}f_{1}+\frac{\partial \phi_{4}}{\partial w_{1}}f_{2}+\frac{\partial \phi_{4}}{\partial \upsilon_{2}}f_{3}+\cdots +\frac{\partial \phi_{4}}{\partial w_{3}}f_{6}\\ \phi_{6}=\frac{\partial \phi_{5}}{\partial \upsilon_{1}}f_{1}+\frac{\partial \phi_{5}}{\partial w_{1}}f_{2}+\frac{\partial \phi_{5}}{\partial \upsilon_{2}}f_{3}+\cdots +\frac{\partial \phi_{5}}{\partial w_{3}}f_{6} \end{array}\right.$$

where ∂Øi=∂xj is the partial derivative of the ith row of the embedding map Ø, with respect to the jth state variable. We obtain the observability matrix by taking the Jacobian of Eq. (A2). In this FN network, the observability matrix is dependent on the state variables and is thus a function of the location in phase space as the system evolves in time. Letellier et al. [28] used averages of the observability index over the state trajectories in phase space as a qualitative measure of observability. We adopt this convention when computing observability of various network motifs. The indices are computed for each time point in the trajectory, and then the average is taken over all of the trajectories.

Constructing the nonlinear controllability matrix for motif 1 from node 1 begins with the control input function g=B**u**(*t*)=1,0,0,0,0,0]^T^ and its Lie bracket with_f_ in Eq. (A1). We respect to the nonlinear vector field exclude the internal driving square wave function here since it is connected to all three nodes, would provide no contribution in the Lie bracket mapping, and we are interested in the mapping from the control input g to the states in order to determine if the system can be controlled:

$$\left[f,g\right]=\underset{0}{\underset{︸}{\frac{\partial g}{\partial x}}}f-\frac{f}{x}g=\left\{ \begin{array}{l} -\frac{\partial f_{1}}{\partial \upsilon_{1}}g_{1}-\cdots -\frac{\partial f_{1}}{\partial w_{3}}g_{6}\\ -\frac{\partial f_{2}}{\partial \upsilon_{1}}g_{1}-\cdots -\frac{\partial f_{2}}{\partial w_{3}}g_{6}\\ -\frac{\partial f_{3}}{\partial \upsilon_{1}}g_{1}-\cdots -\frac{\partial f_{3}}{\partial w_{3}}g_{6}\\ -\frac{\partial f_{4}}{\partial \upsilon_{1}}g_{1}-\cdots -\frac{\partial f_{4}}{\partial w_{3}}g_{6}\\ -\frac{\partial f_{5}}{\partial \upsilon_{1}}g_{1}-\cdots -\frac{\partial f_{5}}{\partial w_{3}}g_{6}\\ -\frac{\partial f_{6}}{\partial \upsilon_{1}}g_{1}-\cdots -\frac{\partial f_{6}}{\partial w_{3}}g_{6} \end{array}\right.$$

where ∂**g**/∂**x**=0 since **g** is the same at each node ∂f_i_=∂x_j_ the partial derivative of the ith row of the nonlinear vector field f(*x*) with respect to the jth stateg. variable, and **g** is the ith component of the input vector **g** We construct the controllability matrix from the definitions in Eqs. (7) and (8), as the control input function g and its higher Lie brackets from$\left(ad_{f}^{\text{1}},g\right)$ to $\left(ad_{f}^{\text{5}},g\right)$ with respect to the nonlinear vector field system equations:

$$Q\text{=}[g\text{,}\left[f\text{,}g\right]\text{,}\left[f\text{,}\left[f\text{,}g\right]\right]\text{,(}ad_{f}^{\text{3}}\text{,}g\text{),(}ad_{f}^{\text{4}}\text{,}g\text{),(}ad_{f}^{\text{5}}\text{,}g\text{)}]$$

2. Observability and controllability index distribution

Log-scaled histograms (Fig. 9) of the index distributions reveal that the local observability (controllability) along the trajectories in phase space are close to a lognormal distribution. After removing zeros from the data, these log-normal distribution fits are computed and verified with the χ^2^ test metric for all of the observability and controllability computation cases that contain an adequate number of data points to accurately compute the fit (over 90% of the data). The χ^2^ test for goodness of fit confirms that the data come from a log-normal distribution with 95% confidence level. This type of zeros-censored log-normal distribution is known as a delta distribution [57], and the estimated mean *κ* and variance *ρ*^*2*^ are adjusted to account for the proportion of data points that are zero, δ, as follows:

$$\delta =\frac{\#\left\{i:x_{i}=0\right\}}{n},\;$$

$$\kappa =(1-\delta )e^{\mu +0.5\sigma^{2}},\;$$

$$\rho^{2}=(1-\delta )e^{2\mu +\sigma^{2}}\left[e^{\sigma^{2}}-(1-\delta )\right]$$

where μ and σ are the mean and variance associated with the log-normal distribution computed from the nonzero data. We use these equations to compute the statistics in the plots in Sec. IV(Figs. 2–7).

3. Group representation analysis of symmetries in motif 1

We examine motif 1 in Fig. 8, which has **S**_**3**_ symmetry. Determined from Eq. (15), there are three classes of group elements C_1_ = {E},C_2_ ={σ_1_;σ_2_;σ_3_}, and C_3_ = {C_3_,$c_{3}^{2}$} Reduction of D(R) yields the two, one-dimensional and one two-dimensional (*l*_1_ = l_2_ = 1,*l*_3_ = 2) irreducible representations [computed from Eq. (16)] D^(1)^(R), D^(2)^(R), and D^(3)^(R) of **S**_**3**_, which are found in Table I and from Eq. (17) appear 1, 0, and 2 times in D(R) , respectively. Forming the generating matrix in Eq. (19), we construct α for motif 1 as follows:

**TABLE I.** Irreducible representations for S3 symmetry.

R	E	σ_1_	σ_2_	σ_3_	C_3_	_$C_{3}^{2}$_
D^(1)^(R)	1	1	1	1	1	1
D^(2)^(R)	1	−1	−1	−1	1	1
D^(3)^(R)	$\left[\begin{array}{cc} 1 & 0\\ 0 & 1 \end{array}\right]$	$\left[\begin{array}{cc} -1 & 0\\ 0 & 1 \end{array}\right]$	$\left[\begin{array}{cc} \frac{1}{2} & -\frac{\sqrt{3}}{2}\\ -\frac{\sqrt{3}}{2} & -\frac{1}{2} \end{array}\right]$	$\left[\begin{array}{cc} \frac{1}{2} & \frac{\sqrt{3}}{2}\\ \frac{\sqrt{3}}{2} & -\frac{1}{2} \end{array}\right]$	$\left[\begin{array}{cc} -\frac{1}{2} & -\frac{\sqrt{3}}{2}\\ \frac{\sqrt{3}}{2} & -\frac{1}{2} \end{array}\right]$	$\left[\begin{array}{cc} -\frac{1}{2} & \frac{\sqrt{3}}{2}\\ -\frac{\sqrt{3}}{2} & -\frac{1}{2} \end{array}\right]$

$$G1\left(1\right)=\sum_{R\in S_{3}}D^{\left(1\right)}(R)11*D(R)I\;$$

$$=1\left[\begin{array}{ccc} 1 & 0 & 0\\ 0 & 1 & 0\\ 0 & 0 & 1 \end{array}\right]+1\left[\begin{array}{ccc} 1 & 0 & 0\\ 0 & 0 & 1\\ 0 & 1 & 0 \end{array}\right]+1\left[\begin{array}{ccc} 0 & 0 & 1\\ 0 & 1 & 0\\ 1 & 0 & 0 \end{array}\right]+\cdots \;\;\;\;\;$$

$$\begin{array}{l} 1\left[\begin{array}{ccc} 0 & 1 & 0\\ 1 & 0 & 0\\ 0 & 0 & 1 \end{array}\right]+1\left[\begin{array}{ccc} 0 & 1 & 0\\ 0 & 0 & 1\\ 1 & 0 & 0 \end{array}\right]+1\left[\begin{array}{ccc} 0 & 0 & 1\\ 1 & 0 & 0\\ 0 & 1 & 0 \end{array}\right] \end{array}$$

$$=\;\left[\begin{array}{ccc} 2 & 2 & 2\\ 2 & 2 & 2\\ 2 & 2 & 2 \end{array}\right]$$

where each linearly independent row of G is a column of α, and thus,

$$\left[\begin{array}{l} 2\\ 2\\ 2 \end{array}\right]\vec{normalize}\left[\begin{array}{l} \frac{1}{\sqrt{3}}\\ \frac{1}{\sqrt{3}}\\ \frac{1}{\sqrt{3}} \end{array}\right]=\left[\begin{array}{l} \alpha_{11}\\ \alpha_{12}\\ \alpha_{13} \end{array}\right]$$

defines the first column of α. We know from Eq. (17) that D^(2)^(R) appears zero times in D(R) and thus yields no contribution to α_. Continuing, we have the last two_ computations from the two-dimensional irreducible representation D^(3)^(one for each row),

$$G_{1}^{\left(3\right)}=\sum_{R\in S_{3}}D^{\left(3\right)}{\left(R\right)}_{11}^{*}D(R)I\;\;\;\;\;\;\;\;\;\;$$

$$=1\left[\begin{array}{ccc} 1 & 0 & 0\\ 0 & 1 & 0\\ 0 & 0 & 1 \end{array}\right]-1\left[\begin{array}{ccc} 1 & 0 & 0\\ 0 & 0 & 1\\ 0 & 1 & 0 \end{array}\right]+\frac{1}{2}\left[\begin{array}{ccc} 0 & 0 & 1\\ 0 & 1 & 0\\ 1 & 0 & 0 \end{array}\right]+\cdots \;\;\;\;\;\;\;\;\;\;$$

$$=\left[\begin{array}{ccc} 1 & 0 & 0\\ 0 & \frac{3}{2} & -\frac{3}{2}\\ 0 & -\frac{3}{2} & \frac{3}{2} \end{array}\right],$$

which after normalization yields

$$\left[\begin{array}{l} 0\\ \frac{3}{2}\\ -\frac{3}{2} \end{array}\right]\vec{normalize}\left[\begin{array}{l} 0\\ \frac{1}{\sqrt{2}}\\ -\frac{1}{\sqrt{2}} \end{array}\right]=\left[\begin{array}{l} \alpha_{21}\\ \alpha_{22}\\ \alpha_{23} \end{array}\right]$$

and

$$G2\left(3\right)=\sum_{R\in S_{3}}D^{\left(3\right)}(R)22*D(R)I\;\;\;\;\;\;\;\;\;\;\;$$

$$=\left[\begin{array}{ccc} 1 & 0 & 0\\ 0 & 1 & 0\\ 0 & 0 & 1 \end{array}\right]+\left[\begin{array}{ccc} 1 & 0 & 0\\ 0 & 0 & 1\\ 0 & 1 & 0 \end{array}\right]-\frac{1}{2}\left[\begin{array}{ccc} 0 & 0 & 1\\ 0 & 1 & 0\\ 1 & 0 & 0 \end{array}\right]+\cdots \;\;\;\;\;\;\;\;\;\;$$

$$=\left[\begin{array}{ccc} 2 & -1 & -1\\ -1 & \frac{1}{2} & \frac{1}{2}\\ -1 & \frac{1}{2} & \frac{1}{2} \end{array}\right]$$

yields the last column of α (after normalization),

$$\left[\begin{array}{c} 2\\ -1\\ -1 \end{array}\right]\vec{normalize}\left[\begin{array}{c} 2\\ -\frac{1}{\sqrt{6}}\\ -\frac{1}{\sqrt{6}} \end{array}\right]=\left[\begin{array}{l} \alpha_{31}\\ \alpha_{32}\\ \alpha_{33} \end{array}\right]\cdot$$

Finally, the coordinate transformation matrix α is

$$\alpha =\left[\begin{array}{ccc} \frac{1}{\sqrt{3}} & \frac{2}{\sqrt{6}} & 0\\ \frac{1}{\sqrt{3}} & -\frac{1}{\sqrt{6}} & \frac{1}{\sqrt{2}}\\ \frac{1}{\sqrt{3}} & -\frac{1}{\sqrt{6}} & -\frac{1}{\sqrt{2}} \end{array}\right]$$

And computation is concluded in Sec. V B.

4. Dilations of the graph of (A,B)

In Ref. [7], the graph G of the pair (*A*,*B*)is defined as a graph of n + 1 nodes *e*_1_; *e*_2_;…; *e*_*n*+1_, where n is the dimension of *A* and *e*_*n*+1_ is called the “origin” (the input).The vertex set *S* = {*e*_1_; *e*_2_,…, *e*_*n*_ is defined as the set of all nodes in G excluding the origin (*e*_*n*+1_ ). A dilation is present in G if and only if |T(S)|<|S| where *T*(*S*) is defined as the set of all nodes that have a directed edge pointing to a node in the set S.
